# Current and Future Strategies in the Diagnosis and Management of Penile Cancer

**DOI:** 10.1155/2011/593751

**Published:** 2011-05-30

**Authors:** Samuel M. Lawindy, Alejandro R. Rodriguez, Simon Horenblas, Philippe E. Spiess

**Affiliations:** ^1^Department of Genitourinary Oncology, H. Lee Moffitt Cancer Center, Tampa, FL 33612, USA; ^2^ Department of Urologic Oncology, Netherlands Cancer Institute and Antoni van Leeuwenhoek Hospital, Plesmanlaan 121, 1066 CX Amsterdam, The Netherlands

## Abstract

Penile cancer is an uncommon malignancy that has a devastating effect on the patient while also being challenging to diagnose and treat. By implementing preventive measures, we can decrease the incidence of this disease and improve the quality of life of our patients. Early detection plays an important role in disease control and proper diagnostic modalities must be used in order to accurately identify the cancer and its progression. Primary penile lesions should be initially approached when surgically feasible and clinically appropriate with penile preserving surgical techniques. Advances in inguinal lymph node detection and management, has improved the clinical outcome of penile cancer. Advanced penile cancer still portends a poor prognosis and should be approached via a multimodal treatment regimen. In this review, we address the importance of prevention, early detection, and the contemporary management of primary penile lesions, as well as the advances in inguinal lymph node disease detection and surgical treatment, for both localized and advanced disease.

## 1. Introduction

Penile cancer is an uncommon disease in the US and Europe that has a devastating effect on the patient while also being challenging to diagnose and treat. A distinction between benign and malignant penile neoplasms must be made in order to offer the most effective treatment [1]. In 2010, the new cases of penile cancer in the United States are about 1,250 with 310 deaths, with an incidence rate of 0.3 to 1.8 per 100,000 [2, 3]. Penile cancer is much more common in African, Asian, and South American countries, constituting about 10% of malignant disease in these countries and thus posing a considerable health concern [1, 4]. Notably, Paraguay and Uganda have an incidence rate of 4.2 and 4.4 per 100,000, respectively [4]. The lowest incidence is found in Israeli Jews (0.1/100,000) [3]. Cancer of the penis most commonly affects men between the ages of 50–70, with only 19% at ages <40 and 7% <30 [3]. Squamous cell carcinoma of the penis was found to be 43% greater in men from countries where the poverty level is >20% [3]. 

## 2. Risk Factors

The presence of an intact foreskin has been identified as an important risk factor for developing penile cancer. Maden et al. [5] found that the risk of penile cancer was 3.2-times greater among men who had never been circumcised relative to men circumcised at birth and 3.0-times greater among men circumcised after the neonatal period [3, 5]. In addition, penile cancer is rarely seen in Jews, as they are circumcised at birth [3]. 

A history of phimosis is also a significant risk factor. 25–60% of patients who had a history of phimosis develop penile cancer [3]. Hellberg et al. [6] performed a retrospective study of 244 men with penile cancer and 232 matched controls. The relative risk of penile cancer among men with phimosis was 64.6 [3, 6]. Phimosis leads invariably to retention of the normally desquamated epidermal cells and urinary products (smegma) resulting in conditions of chronic irritation with or without bacterial inflammation of the prepuce and the glans [4]. The frequency of phimosis in men with penile carcinoma is high, ranging from 44% to 85% [4, 7]. However, there is no supporting evidence of the role of smegma as a carcinogen and is, therefore, not believed to contribute to the development of penile cancer.

Several studies have also identified smoking as an associated risk factor for the development of penile cancer [3]. Balanitis and penile injury has also been found to increase the risk of penile cancer [3, 5, 6]. Number of sexual partners and history of genital warts or other sexually transmitted disease may also play a part in the risk for developing cancer of the penis [4, 8]. HPV types 16 and 18 have a strong correlation with penile carcinoma, reported as 25–94.7% of cases with type 16 and 10.5–55.4% with type 18 [3, 5, 9]. Review of the literature revealed that about 45–80% of penile cancers are related to HPV [4, 7, 8, 10, 11]. 

### 2.1. Goal

The goal of this review is to highlight the current practices in prevention, detection, and treatment for primary penile lesions as well as advanced penile cancer with an emphasis on recent data and future prospects for the management and diagnosis of penile carcinoma. 

## 3. Prevention

### 3.1. Circumcision

As stated previously, there is much evidence pointing to the association of an intact foreskin with the development of penile cancer. Circumcision in early childhood could prevent phimosis and other risk factors, such as HPV infection, thereby reducing the risk of developing penile cancer [4, 12]. Multiple studies have shown that the vast majority of men with penile carcinoma are uncircumcised [12]. In well-developed countries where the incidence of penile cancer is low, this preventive measure may not be necessary. However, other less developed countries with poor hygiene may attain a greater and much more tangible benefit with circumcision [12]. In addition, circumcised men with female partners known to have cervical intraepithelial neoplasia (CIN) had a lower rate of developing PIN compared to men who were uncircumcised [13, 14]. Unfortunately, the advantage of adult circumcision in the prevention of penile cancer has not been well established. 

### 3.2. HPV

Since HPV plays a major role in the development of penile carcinoma, it is a prime target for prevention. Thus far, there are two different types of HPV vaccines: GARDASIL, a quadrivalent vaccine that targets types 6, 11, 16, and 18; and Cervarix, a bivalent vaccine which targets HPV types 16 and 18 [12]. It has been shown that the seroconversion after vaccine is at 99.1–100% with up to 5 years of protection after vaccination in boys 9–15 years old [15, 16]. Unfortunately, long-term data are not yet available and thus can only be assumed that male vaccination of HPV can prevent associated penile cancer development [12]. Additionally, condom use has been shown to effectively reduce the risk of developing genital warts among partners [17]. 

Although there is evidence that suggests that there is a link between smoking and penile cancer [5], the impact of smoking cessation would not likely alter the incidence and management of penile cancer. There is no clear evidence between the connection of poor hygiene and the development of penile cancer. However, the greater incidence of penile carcinoma in third-world countries and the currently available data suggest that poor genital hygiene may play a role in the development of penile cancer [3, 4, 12, 18]. Psoralen Plus Ultraviolet Light A (PUVA) treatment for psoriasis has been shown to increase the incidence of squamous cell carcinoma of the penis by 286-times that of the general population [19, 20]. Therefore, patients undergoing this kind of therapy should be advised to use genitalia shielding as well as close observation for any penile lesions [12]. 

## 4. Early Detection and Diagnosis

Penile carcinoma usually presents with a visible or palpable lesion on the penis [21]. It can also be associated with pain, discharge, bleeding, or foul odor [21]. It has been shown that the glans penis is the most common site for primary disease [22]. There is a significant delay between initial symptoms and seeking medical attention. Narayana et al. [23] showed that only 48% of men sought treatment after 6 months with symptoms, 21% waited beyond 6 months to up to a year, and 30% waited for over a year before seeking medical attention. 

### 4.1. Staging

Based on changes proposed in 2009 by the AJCC, the 2010 TNM staging system was revised as shown in Table 1 [21].

A high index of suspicion for penile cancer must be maintained in men with lesions on the penis, especially if there are known risk factors present. A careful history and examination are key to help characterize the lesion and determine further diagnostic steps. The physical exam can be supplemented by ultrasonography in order to more accurately estimate lesion size and anatomic relations to the tunica albuginea, corpus cavernosum, and urethra [21, 24–26]. Moreover, the 2004 European Association of Urology (EAU) identified penile ultrasound as the initial diagnostic test of choice in determining depth of tumor penetration [27, 28]. 

CT imaging is not considered an imaging modality of choice for penile cancer due to its poor visualization of penile tissue planes and thus ineffective in evaluating the tumor stage of the cancer [27]. In a small study, the combination of PET/CT imaging was shown to have a sensitivity and specificity of 75% for detecting primary penile tumors [29], but remained to be ineffective in determining tumor stage [27]. Cavernosography has been shown to successfully stage patients [30]; however, it is limited in its ability to evaluate tumor beyond the corporal bodies [27].

MRI has been shown to provide adequate staging capabilities when combined with pharmacologically induced penile erection [31]. It is the most sensitive imaging modality for penile carcinomas due to its superior soft tissue contrast and assessment of penile fascial planes [27]. In addition, endoluminal coils may also be used to enhance the MRI images [32].

The AJCC states that a pathologic diagnosis must be made, requiring the use of either punch, excisional, or incisional biopsy [21, 33]. 

## 5. Primary Penile Lesions

The surgical management of a malignant penile lesion depends on the grade and stage of the disease [34]. The gold standard treatment for primary penile lesions remains to be total or partial penectomy. This standard therapy of total/partial penectomy for penile cancer achieves local control rates above 90% but also causes significant disfiguration, leading to loss of function and psychosexual morbidity [35]. 

However, new evidence has shown that a 5–10 mm margin is as safe as a 2 cm surgical margin [36] and has been recommended by the EAU [35, 37]. It has been shown that these smaller margins of 10–20 mm provide adequate tumor control [36, 38, 39]. 

### 5.1. CIS

First line therapy for carcinoma in situ (Tis) is with topical chemotherapy using 5% 5-fluorouracil (5-FU) cream for 6 weeks. Studies have shown good sustained response rates at 5 yrs [40]; however, more studies are needed to confirm this data as well as the use of Imiquimod as second-line therapy [34, 41]. CO_2_ and Nd-YAG lasers have also proved to be effective in the treatment of Tis [42], with no significant differences in recurrence and survival among patients with partial penectomy, radiotherapy, or laser therapy [43]. However, its use in T2 disease has been shown to increase the risk of nodal spread [44], thus stressing the importance of proper staging, management, and followup for the different types of penile cancer. 

Intractable CIS may be managed surgically by a total glans resurfacing technique used for Balanitis xerotica obliterans [45] that has proven to be effective for CIS [34, 46]. Circumcision is also effective for prepuce lesions; however, glans removal and reconstruction offer better protection [35]. Complete glansectomy may be required if urethra is involved [35]. Studies have shown that recurrence rates for these conservative surgeries are between 3.1–31.4% [35]. Tumors less than 1 cm in size are ideal for Mohs micrographic surgery [35], and can provide maximal chance of normal tissue preservation and function [47]. 

### 5.2. T1

Wide local excision with primary closure of the glans is appropriate if there is no urethral involvement [34]. Larger lesion may require split skin or full thickness graft to cover the defect. Recurrence rates are reported to be at 50% after 2 years [48], thus requiring careful patient selection and close surveillance [34]. Bandieramonte et al. [49] studied a total of 224 patients with T1 disease who underwent CO_2_ laser excision for penile carcinoma treatment. Complete excision was achieved in 98.7% at lateral margins and 96.9% in the deep margins. The study had a 10-year recurrence rate of 17.5% and a 10-year amputation rate of 5.5%, concluding that early stage penile carcinoma can be treated with organ-sparing laser therapy [49]. 

### 5.3. T2

The new TNM staging system does not differentiate between T2a with invasion into the corpus spongiosum, and T2b with tunica or corpora cavernosa invasion. Studies suggest that a higher rate of lymph node metastasis is seen in T2b cavernosum invasion [35]. If disease is limited to the glans, total glansectomy with excision of the glans penis from the corpora cavernosa is appropriate [34]. Involvement of the tunica albuginea and/or corpora cavernosa by the cancer rules out conservative surgery [35]. In most cases, partial penectomy is the appropriate therapy and total penectomy with phalloplasty is warranted if the tumor extends proximally [34]. 

### 5.4. Radiotherapy

Usually indicated for T1-T2 tumors of less than 4 cm and delivered via external beam radiotherapy or brachytherapy [35]. Studies have shown external beam radiotherapy to have a 5-year overall survival rate of 88%, but at the same time carries a higher relapse rate than penectomy [50]. Brachytherapy has an overall survival of 69–78% with preservation of the penis in 86% of cases [35, 51]. However, external beam radiotherapy and brachytherapy are associated with severe complications down the line, such as urethral stenosis, telangiectasia, fibrosis/atrophy, and penile necrosis resulting in amputation [35, 51, 52]. EAU guidelines for radiotherapy (including brachytherapy) limits its use for only T1-T2 tumors of the glans or sulcus and only palliative external beam radiation for locally advanced metastatic disease [35, 37]. Chronic ulcers or nonhealing areas after radiotherapy should be considered tumor recurrence until proven otherwise [34, 53]. 

### 5.5. Chemotherapy

There are no large randomized trials concerning chemotherapy comparing one regimen versus another. Neoadjuvant chemotherapy has shown promising results with high response rates and pathological remissions with combination regimens such as bleomycin, methotrexate, and cisplatin [35]. Agents targeting angiogenesis or COX-2 pathways may hold promise in treating penile cancer [35]. Specifically, cetuximab has emerged as an effective therapy option for head and neck squamous cell carcinoma. Recent data has shown cetuximab to provide significant improvement in regional control and overall survival [54]. There are several ongoing trials with cetuximab and its efficacy on head and neck SCC that have thus far shown promising results [55]. 

## 6. Inguinal Lymph Nodes

Inguinal lymph node dissection (ILND) is standard practice for patients with lymph node metastasis or patients with high-risk primary penile tumors (T2 or greater, >50% differentiated tumor) [56]. Unfortunately, complications are common and can have significant morbidity, such as lymphedema, skin necrosis, and hematoma [56]. Therefore, it is important to accurately determine the patients that are in need of ILND via imaging, biopsy, and minimally invasive techniques if metastasis is detected [57].

The presence and degree of lymph node involvement in penile cancer plays a major role in prognosis [27]. Initial assessment is by physical exam. About 50% of patients with penile cancer have palpable lymphadenopathy, with only half of these actually turning out to be nodal metastases [27]. The use of antibiotics for a 2–6 week period has become common practice in order to distinguish between metastatic disease or inflammatory nodal response [27]. It has been shown that palpable lymph nodes that remain after antibiotic treatment have 90% chance of metastatic spread [27, 58]. There is evidence to suggest that it may be more beneficial to undergo FNA cytology to evaluate palpable inguinal lymphadenopathy for metastatic penile cancer without the need for prolonged antibiotic treatment [59].

**Figure 1:**
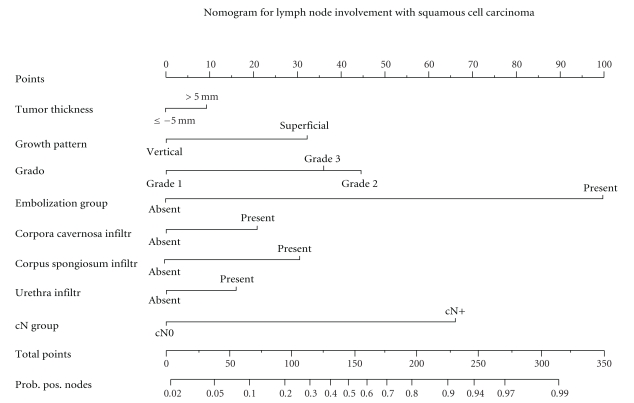
Nomogram predicting the probability of lymph node metastasis for penile cancer. Reprinted with permission from: Ficarra et al. [60].

Nomogram developed by Ficarra et al. [60] for predicting probability of lymph node involvement shown in Figure 1.

High-resolution ultrasound and color Doppler may be used to assess palpable lymph nodes via detecting abnormalities in architecture and vascularity [61]. With color Doppler, it has been noted that metastatic nodes show peripheral vascularity while reactive nodes show a hilar perfusion pattern [27, 61]. But the sensitivity and specificity of this study may be inadequate unless combined with fine-needle aspiration cytology [27, 62]. Combination of FNA with US has shown to have 40% sensitivity and 100% specificity [63], and has been suggested as the initial investigation for clinically palpable nodes in patients with high risk of lymph nodes metastases [64].

Cross-sectional imaging such as CT and MRI rely on changes in size and thus have been proven to be ineffective in the evaluation of lymph node metastases [27, 64]. PET/CT has been shown to have a high diagnostic accuracy with 90% sensitivity and 100% specificity in the setting of cytologically proven inguinal node metastases [65], but is inadequate in patients with nonpalpable nodes [66].

Lymphotropic nanoparticle-enhanced MRI (LNMRI) is a new and promising noninvasive imaging study for lymph node staging. LNMRI was shown to have a sensitivity of 100% and a specificity of 97% in a recent small study [67]. In the same study, it was shown that LNMRI can accurately detect lymph node metastasis even in patients without palpable inguinal nodes [27, 67]. Thus, this imaging modality may be effective in determining whether a patient should undergo unilateral versus bilateral ILND [27]. Unfortunately, this study is not widely available and requires lengthy interpretation by the radiologist. Moreover the contrast used has not been FDA approved and is not available in Europe anymore [27]. 

Diffusion-weighted MRI is another imaging modality that provides structural information about different tissues. When combined with LNMRI, it provides an effective and faster tool for the evaluation of inguinal lymph node metastases [24, 68].

Dynamic sentinel lymph node biopsy (DSNB) has been established as the modality of choice for nodal evaluation in patients with melanoma [27]. Initial studies of DSNB in the evaluation for penile cancer nodal spread revealed high false-negative rates between 22–77% [27]. However, after certain technical modifications this high false-negative rate dropped [27]. A recent study of DSNB combined with ultrasound-guided FNA revealed a negative predictive value of 100% [27, 62]. Also, Leijte et al. [69] found DSNB to have a sentinel node identification rate of 97% with a false negative rate of 7%.

### 6.1. Inguinal Lymph Node Dissection

Inguinal lymph node dissection (ILND) can have significant morbidity [56]. Therefore, it is important to accurately determine the patients that are in need of ILND via imaging, biopsy, and minimally invasive techniques if metastasis are detected [57].

Modified (limited) ILND consists of a shortened skin incision with preservation of saphenous vein and subcutaneous tissues superficial to Scarpa's fascia; no dissection lateral to femoral artery or caudal to the fossa ovalis, without Sartorius muscle transposition [64, 70]. However, it has been shown that modified ILND has up to a 5.5% risk of leaving occult metastasis [64, 71]. Radical (complete) ILND is described by Daseler et al. [72] and can be curative if metastasis is limited to the inguinal nodes [64]. 

Video endoscopic inguinal lymphadenectomy (VEIL) is a new minimally invasive procedure that can result in lower complication rates and shorter hospitalization stay [64] without compromising oncologic results [35, 73]. A recent study revealed that VEIL had a morbidity rate of only 15% while standard open ILND had a 70% morbidity rate [73]. In addition, the number of nodes removed was the same for VEIL as it is for open ILND, while also showing no local or systemic relapse after a median followup of 33 months [35, 73]. However, data is still lacking in this promising new treatment modality for ILND and would benefit from larger multicenter trials with an extensive follow-up period.

The question remains whether or not bilateral ILND is warranted when unilateral disease is discovered. Studies have shown that presence of positive nodes on one side has an incidence of positive nodes on the contralateral side of about 20–60% [64, 74]. More recently, it has been suggested that with greater than 2 inguinal lymph node metastasis, there is 30% of occult contralateral involvement [64, 75]. 

On the other hand, a recent study revealed that the size of the sentinel lymph node during DSNB was the only significant prognostic variable for additional lymph node involvement, with a node of less than 2 mm to suggest no additional nodal involvement, and thus sparing contralateral ILND [64, 76]. Pelvic lymph node involvement and the need for PLND is controversial with variable data on the matter [64]. Studies range from recommending PLND based on grade of primary tumor, to number of positive nodes in ILND [64]. The involvement of Cloquet's node has also been studied to predict the likelihood of pelvic lymph node spread and the need for PLND [64]. A recent study showed that lymph node metastasis in Cloquet's node has a sensitivity of 30% and specificity of 94% for pelvic lymph node involvement [64, 77]. 

## 7. Advanced Penile Cancer

Advanced primary penile cancer with bulky adenopathy (>3 cm) warrants penectomy with groin dissection [78]. If nodes are unresectable, a multimodal approach would provide the best cancer control [78]. Neoadjuvant chemotherapy may be effective in downsizing the initially inoperable metastasis to allow for surgical removal [52]. Agents such as bleomycin, methotrexate, 5-FU, ifosfamide, and cisplatin each alone have a response rate of only 20% [52, 79, 80]. Nevertheless, combination regimens of these chemotherapy agents have yielded greater response rates of 25–72% [52, 81]. 

The combination of bleomycin, methotrexate, and cisplatin have been studied in a prospective clinical trial that revealed an overall response rate of 32.5% with a median response duration of 16 weeks and median survival time of 28 weeks [78, 82]. The high toxicity levels and degree of efficacy for this combination suggest that this may be a poor choice for adjuvant or neoadjuvant chemotherapy [78, 82]. Recent studies have shown promising data that a combination neoadjuvant chemotherapy of paclitaxol, ifosfamide, and cisplatin prior to surgery is well tolerated and had significant response in patients with bulky lymph node metastasis with 36.7% of patients remaining free of recurrence [83–85]. It has also been reported that adjuvant chemotherapy with a combination of cisplatin, 5-FU, and docetaxol would be beneficial in advanced disease [52]. Recent EUA practice guidelines advocate for neoadjuvant chemotherapy for locally advanced disease using a combination regimen with follow-up surgical resection [37, 86]. 

Radiotherapy may also play an important role in the initial treatment of unresectable lymph node metastasis [78]. Much information regarding squamous cell carcinoma of the penis is better understood with larger studies regarding SCC of the vulva and anal canal [78]. Several large randomized studies have shown that the combination of chemoradiotherapy with surgical treatment is an effective and well-tolerated management for anal and vulvar cancer [78]. Thus far, there have been no large randomized trials for penile cancer. Thus, extrapolation of this data into the realm of penile carcinoma management would suggest that inoperable advanced penile cancer may best be treated initially with chemoradiotherapy followed by nodal dissection [78]. 

The relationship between epidermal growth factor receptor (EGFR) and squamous cell carcinoma in other areas, such as the head and neck, have been well studied resulting in targeted agents against the receptor with improved survival and response to radiotherapy [54, 86]. A recent retrospective study by Carthon and colleagues [87] revealed the use of erlotinib alone, cetuximab, or cetuximab plus cisplatin had an overall survival range of 2.8 months to 48 months with median time to disease progression in the range of 0.37 to >37 months [86]. The potential merit of neoadjuvant chemotherapy plus/minus targeted therapy with EGFR inhibitors followed by consolidative surgical resection is of significant clinical interest, and we await results of planned phase II prospective trials in this regard. 

## 8. Conclusions

Penile carcinoma is rare in the United States and Europe, but is an important cause of morbidity and mortality in many other countries [1–4]. Therefore, it is important to implement preventative measures to decrease the incidence of disease and improve quality of life [4, 12]. 

Early detection plays a vital role in disease control and the proper diagnostic modalities must be used in order to accurately identify the cancer and its progression. The use of imaging in combination with biopsy is an effective means in determining disease stage and grade [21, 27, 33].

 Primary penile lesions should be initially approached with penile-preserving modalities including Mohs, CO_2_ and Nd-YAG lasers, and chemo/brachytherapy [34, 35]. ILND should be undertaken after proper assessment for metastatic spread to the nodes via DSNB in combination with FNA cytology [27, 62, 69]. The use of LNMRI has shown promise as a new tool for detecting lymph node metastasis and presents as possible standard future approach to the evaluation of inguinal lymph nodes [27, 67]. The degree of ILND depends greatly on the extent of lymph node involvement and grade [27]. VEIL has proven to be an effective treatment option for ILND with a superior complication rate versus open nodal dissection [35, 64, 73]. Further data regarding its use and the different approaches to metastatic lymph node disease management is essential and would greatly benefit the advancement of penile cancer care.

Advanced penile cancer holds a poor prognosis and must be approached via a multimodal treatment regimen that includes neoadjuvant chemotherapy followed by surgical resection [78]. In select cases, the use of radiotherapy has been shown to be effective in the reduction of bulky nonresectable disease to allow for surgical removal [78]. The chemotherapy combination that has so far shown to be the most effective and well tolerated is the paclitaxol, ifosfamide, and cisplatin combination [37, 78, 83–86]. However, large randomized trials are lacking in this area and would shed light as to the best therapy for advanced penile cancer. 

## Figures and Tables

**Table 1:** TNM staging system for penile cancer.

Definitions of TNM		
Primary tumor (T)		

TX	Primary tumor cannot be assessed	
T0	No evidence of primary tumor	
Tis	Carcinoma in situ	
Ta	Noninvasive verrucous carcinoma	
T1a	Tumor invades subepithelial connective tissue without LVI and is not poorly differentiated (i.e., G3-4)	
T1b	Tumor invades subepithelial connective tissue with LVI or is poorly differentiated	
T2	Tumor invades corpus spongiosum or cavernosum	
T3	Tumor invades urethra	
T4	Tumor invades other adjacent structures	

Regional lymph nodes (N)		

	Clinical stage definition	Pathologic stage definition
NX	Regional lymph nodes cannot be assessed	Regional lymph nodes cannot be assessed
N0	No palpable or visibly enlarged inguinal lymph nodes	No regional lymph node metastasis
N1	Palpable mobile unilateral inguinal lymph node	Metastasis in a single inguinal lymph node
N2	Palpable mobile multiple or bilateral inguinal lymph nodes	Metastasis in multiple or bilateral inguinal lymph nodes
N3	Palpable fixed inguinal nodal mass or pelvic lymphadenopathy unilateral or bilateral	Extranodal extension of lymph node metastasis or pelvic lymph node or lymph nodes unilateral or bilateral

Distant Metastasis (M)		

M0	No distant metastasis	
M1	Distant metastasis	

Reprinted with permission from Barocas and Chang [21].
